# Association between serum 25‐hydroxyvitamin D level and inflammatory markers in hemodialysis‐treated patients

**DOI:** 10.1002/iid3.1201

**Published:** 2024-04-23

**Authors:** Chunlei Luo, Xueyan Bian, Lingling Bao, Qingqing Xu, Chunyang Ji

**Affiliations:** ^1^ Department of Nephrology The First Affiliated Hospital of Ningbo University Ningbo Zhejiang China

**Keywords:** 25‐hydroxyvitamin D, hemodialysis, inflammatory markers

## Abstract

**Objective:**

To investigate the relationship between serum 25‐hydroxyvitamin D (25(OH)D) level with novel inflammatory markers in hemodialysis‐treated patients.

**Methods:**

A total of 167 maintenance hemodialysis‐treated patients were enrolled in this cross‐sectional study. The patients were divided into vitamin D deficiency (a serum 25(OH)D level <20 ng/mL) and nondeficiency (a serum 25(OH)D level ≥20 ng/mL) groups. The neutrophil to lymphocyte ratio (NLR), platelet to lymphocyte ratio (PLR), and monocyte to lymphocyte ratio (MLR) were calculated by the complete blood cell count. The relationship between 25(OH)D level with other parameters was assessed by bivariate correlation analysis and linear regression analysis.

**Results:**

There were significant differences between the two groups in terms of age, diabetes, levels of albumin, creatinine, high‐density lipoprotein cholesterol (HDL‐C) and low‐density lipoprotein cholesterol (LDL‐C) as well as NLR and MLR (*p* = .004, *p* = .031, *p* < .001, *p* = .043, *p* = .008, *p* = .006, *p* = .002, and *p* < .001, respectively). There exist negative correlations between serum 25(OH)D level with age, diabetes, alkaline phosphatase level, NLR, PLR, and MLR (*p* = .002, *p* = .002, *p* = .037, *p* = .001, *p* = .041, and *p* < .001, respectively) and positive correlations between serum 25(OH)D level with albumin level, creatinine level, phosphorus level, HDL‐C, and LDL‐C (*p* < .001, *p* < .001, *p* = .013, *p* = .02, *p* = .002, respectively). Multiple analysis results showed that sex, diabetes, albumin level and NLR were independently associated with serum 25(OH)D level (*p* = .021, *p* = .015, *p* = .033, and *p* = .041, respectively). High values of NLR and MLR were associated with patients with serum 25(OH)D deficiency. There were negative interplays between serum 25(OH) D level with NLR, PLR, and MLR and also an independent association between serum 25(OH) D level with NLR.

**Conclusion:**

Collectively, serum 25(OH)D level has a negative correlation with inflammatory markers.

## 1 INTRODUCTION

Patients with chronic kidney disease (CKD) including end‐stage renal disease (ESRD) are prone to vitamin D deficiency. The prevalence of vitamin D deficiency or insufficiency can reach over 80%.^1^, ^2^ Of note, vitamin D deficiency is implicated in cardiovascular events and all‐cause mortality in hemodialysis‐treated patients.^3^, ^4^ The mortality risk in patients with CKD is decreased by 14% for every 10 ng/mL increase in 25‐hydroxyvitamin D (25(OH)D) level,^5^ while being increased by 30% for 25(OH)D level lower than 18 ng/mL.^6^

Microinflammation occurring in ESRD patients causes atherosclerosis, malnutrition and cardiovascular disease, and is responsible for increased mortality of these patients.^7^ In the context of the confirmed relationship between vitamin D and inflammation, a clear consensus has not been reached. The neutrophil to lymphocyte ratio (NLR), platelet to lymphocyte ratio (PLR), and monocyte to lymphocyte ratio (MLR) are accessible, inexpensive, and reproducible markers for detecting inflammation recently. Neutrophils are the main effector cells during the systemic inflammatory response, which, together with other inflammatory cells, can be used to mediate the early stage of infection as having proinflammatory properties, and NLR is usually characterized by an increase in neutrophils and a decrease in lymphocytes.^8^ Platelets are rich in proinflammatory agents, and the interaction of platelets with T lymphocytes mediated by P‐selectin reduces lymphocyte proliferation, leading to diminished proinflammatory cytokines such as interferon‐α (IFN‐α), tumor necrosis factor‐α (TNF‐α), and interleukin (IL)‐17, and elevated anti‐inflammatory cytokines such as IL‐10.^9^ Besides, increased MLR is strongly associated with the risk of new‐onset CKD in people.^10^ However, there are limited data on the association between vitamin D and these novel inflammatory markers such as NLR and PLR, and no studies evaluating the interplay between vitamin D and MLR in hemodialysis‐treated patients. Therefore, this study concentrated on investigating the correlation between serum 25(OH)D level and novel inflammatory markers in hemodialysis‐treated patients.

## 2 MATERIALS AND METHODS

### 2.1 Study design

This was a cross‐sectional study performed on 187 ESRD patients treated with conventional maintenance hemodialysis from January 2022 to December 2022 in our hospital. The inclusion criteria were patients aged over 18 and receiving treatment of maintenance hemodialysis (4‐h dialysis regimens for twice to thrice per week) for at least 3 months. The exclusion criteria were set as follows: patients having no data on serum 25(OH)D level but a history of malignancy, receiving peritoneal dialysis, accompanied with acute infection (within 2 weeks before enrollment, whether controlled or not) and active inflammatory disease, and receiving treatment of immunosuppressive drug. Overall, 20 patients were excluded, involving five without data of serum 25(OH)D level, two receiving treatment of peritoneal dialysis, six with acute infection, four with malignancy, two with active inflammatory disease, and one being administered with immunosuppressive drug. Ultimately, the data of 167 patients who met the criteria were included in the study, which was approved by the ethics committee of the First Affiliated Hospital of Ningbo University (2023‐042RS) and abided by the Declaration of Helsinki.

### 2.2 Data collection

Clinical and demographic characteristics of the patients (age, sex, presence of diabetes mellitus, body mass index (BMI), dialysis duration, type of vascular access, dialysis frequency and Kt/v index) were recorded at the time of enrollment.

Fasting venous blood samples were collected directly from patients before connection of the dialysis tube by hemodialysis specialist nurse on dialysis day, and then transported to the central laboratory of the hospital immediately for laboratory analysis.

Laboratory data including serum 25(OH)D level, complete blood count with automated differential counts (involving white blood cell [WBC], neutrophils, lymphocytes, and monocytes), levels of high‐sensitivity C‐reactive protein (hsCRP), hemoglobin, albumin, creatinine, calcium, phosphorus, intact parathyroid hormone, and lipid parameters were obtained from the medical record database. NLR, PLR, and MLR were calculated.

According to the Kidney Disease Outcome and Quality Initiative guidelines, vitamin D deficiency is defined as a serum 25(OH)D level <20 ng/mL, insufficiency as a serum 25(OH)D level from 20 to 30 ng/mL, and sufficiency as a serum 25(OH)D level >30 ng/mL.^11^ Therefore, patients in this study were separated into vitamin D deficiency group (a serum 25(OH) D level of <20 ng/mL) and nondeficiency group (a serum level of ≥20 ng/mL).

### 2.3 Statistical analysis

Statistical analyses were carried out using the SPSS 26.0 (IBM Corp.). Descriptive statistics were determined for each variable. Mean ± standard deviation was used to describe continuous variables having normal distribution and median (P25, P75) with asymmetric distribution. The independent samples *t*‐test and the Mann–Whitney *U* test were applied for analyzing continuous variables. Categorical variables analyzed by *χ* ^2^ test were expressed as percentages. Associations between the variables were explored using Pearson's rho (for normally distributed variables) and Spearman's rho (for asymmetrically distributed variables). Linear regression analysis was employed to define independent variables associated with serum 25(OH)D level. A *p* < .05 was considered statistically significant.

## 3 RESULTS

A total of 167 maintenance hemodialysis‐treated patients were included in this study, with the median age of 66 (55–76) years, and 65.3% (*n* = 109) of males. The mean 25(OH)D level was 25.16 ± 10.08 ng/mL. There were 56 (33.5%) patients in vitamin D deficiency group and 111 (66.5%) patients in nondeficiency group. Only 49 patients (29.3%) had a sufficient vitamin D level. The mean serum 25(OH)D level was 14.88 ± 3.56 ng/mL in the deficiency group and 30.34 ± 8.14 ng/mL in the nondeficiency group. The demographics as well as the clinical and laboratory characteristics of the two groups are displayed in Table 1. There were statistically prominent differences between two groups in terms of age, diabetes, levels of albumin, creatinine, high‐density lipoprotein cholesterol (HDL‐C) and low‐density lipoprotein cholesterol (LDL‐C) as well as NLR and MLR (*p* = .004, *p* = .031, *p* < .001, *p* = .043, *p* = .008, *p* = .006, *p* = .002, and *p* < .001, respectively). Although PLR was higher in patients with 25(OH)D deficiency, the difference was not statistically significant (*p* = .075). hsCRP and other parameters were barely different in the two groups.

**Table 1:** Demographics and clinical and laboratory parameters of hemodialysis‐treated patients according to serum 25(OH)D levels.

Parameters	25(OH)D < 20 ng/mL (*n* = 56)	25(OH)D ≥ 20 ng/mL (*n* = 111)	*p*‐Value
Age (years)	72.0 (60.0, 78.8)	65.0 (53.3, 72.8)	.004
Sex, male (*n* [%])	34 (60.7%)	76 (67.6%)	.38
Body mass index (kg/m^2^)	22.07 ± 3.56	22.08 ± 3.46	.99
Diabetes (*n* [%]]	25 (44.6%)	31 (27.9%)	.031
Dialysis duration (months)	25.0 (13.0, 55.8)	23.5 (11.0, 48.0)	.594
Vascular access, fistula or graft (*n* [%])	44 (78.6%)	93 (83.8%)	.407
Conventional maintenance hemodialysis, thrice weekly (*n* [%])	42 (75.0%)	90 (81.1%)	.362
Kt/v index	1.43 ± 0.24	1.44 ± 0.28	.731
25(OH)D (ng/mL)	14.88 ± 3.56	30.34 ± 8.14	<.001
WBC (×10^3^/mm^3^)	5.73 ± 1.94	5.51 ± 1.54	.48
Hemoglobin (g/L)	109.09 ± 14.14	110.66 ± 13.42	.485
Platelet (×10^3^/mm^3^)	168.7 ± 49.22	179.56 ± 53.28	.211
Albumin (g/L)	35.24 ± 3.14	37.32 ± 3.22	<.001
Alkaline phosphatase(U/L)	98.11 ± 44.74	92.78 ± 46.65	.482
Creatinine (μmol/L)	756.46 ± 330.33	856.04 ± 279.35	.043
Phosphorus (mmol/L)	1.51 ± 0.52	1.63 ± 0.41	.13
Calcium (mmol/L)	2.29 ± 0.19	2.30 ± 0.20	.761
iPTH (pg/mL)	123.18 (67.95, 266.81)	160.46 (75.00, 262.50)	.581
Triglyceride (mmol/L)	1.7 ± 1.24	1.9 ± 1.56	.434
HDL‐C (mmol/L)	0.91 ± 0.23	1.03 ± 0.3	.008
LDL‐C (mmol/L)	2.12 ± 0.71	2.47 ± 0.8	.006
Hs‐CRP (mg/L)	1.21 (0.50, 3.40)	0.78 (0.50, 2.45)	.482
Hs‐CRP < 3 mg/L (*n* [%])	40(71.4%)	85 (76.6%)	.469
NLR	4.58 ± 2.22	3.51 ± 1.49	.002
PLR	186.86 (133.32, 234.64)	158.53 (117.01, 198.14)	.075
MLR	0.57 ± 0.28	0.42 ± 0.16	<.001

Abbreviations: 25(OH)D, 25‐hydroxyvitamin D; HDL‐C, high‐density lipoprotein cholesterol; hs‐CRP, high‐sensitivity C‐reactive protein; iPTH, intact parathyroid hormone; LDL‐C, low‐density lipoprotein cholesterol; MLR, monocyte to lymphocyte ratio; NLR, neutrophil to lymphocyte ratio; PLR, platelet to lymphocyte ratio; WBC, white blood cells.

The correlations between 25(OH)D and other parameters were determined using bivariate correlation analysis (Table 2). There exist obvious positive correlations between 25(OH)D with levels of albumin, creatinine, phosphorus, HDL‐C and LDL‐C (*p* < .001, *p* < .001, *p* = .013, *p* = .02, *p* = .002, respectively), and negative associations between 25(OH)D with age, diabetes, alkaline phosphatase level, NLR, PLR, and MLR (*p* = .002, *p* = .002, *p* = .037, *p* = .001, *p* = .041, and *p* < .001, respectively). 25(OH)D had an insignificant association with hsCRP, HB, and WBC. In addition, NLR, PLR, and MLR were positively correlated with hsCRP (*r* = .308, *p* < .001; *r* = .277, *p* < .001; and *r* = .303, *p* < .001, respectively).

**Table 2:** Bivariate correlations between serum 25(OH)D level with other parameters.

Variables	*r*‐Value	*p*‐Value
Sex (male)	.144	.064
Age (years)	−.234	.002
Diabetes	−.237	.002
WBC (×10^3^/mm^3^)	−.134	.085
Hemoglobin (g/L)	−.013	.871
Platelet (×10^3^/mm^3^)	.005	.952
Albumin (g/L)	.281	<.001
Alkaline phosphatase (U/L)	−.162	.037
Creatinine (μmol/L)	.299	<.001
Phosphorus (mmol/L)	.192	.013
Calcium (mmol/L)	.045	.567
iPTH (pg/mL)	.021	.790
Triglyceride (mmol/L)	.05	.531
HDL‐C (mmol/L)	.185	.020
LDL‐C (mmol/L)	.248	.002
Hs‐CRP (mg/L)	−.15	.053
NLR	−.278	.001
PLR	−.160	.041
MLR	−.311	<.001

Abbreviations: 25(OH)D, 25‐hydroxyvitamin D; HDL‐C, high‐density lipoprotein cholesterol; hs‐CRP, high‐sensitivity C‐reactive protein; iPTH, intact parathyroid hormone; LDL‐C, low‐density lipoprotein cholesterol; MLR, monocyte to lymphocyte ratio; NLR, neutrophil to lymphocyte ratio; PLR, platelet to lymphocyte ratio; WBC, white blood cells.

Univariate analysis results identified that sex and levels of albumin, creatinine and LDL‐C were positively correlated to 25(OH)D level (*p* = .024, *p* < .001, *p* = .003, and *p* = .012, respectively), while age, diabetes, NLR, and MLR were negatively correlated to 25(OH)D level (*p* = .002, *p* = .002, *p* < .001, and *p* < .001, respectively). PLR was irrelevant to 25(OH)D level. Multiple analysis data showed that sex, diabetes, albumin level, and NLR were independently associated with serum 25(OH)D level (*p* = .0121, *p* = .015, *p* = .033, and *p* = .041, respectively) (Table 3).

**Table 3:** Correlations between serum 25(OH)D level with other relevant parameters.

Variables	Univariate		Multivariate	
	*β* ± SE (95% CI)	*p*‐Value	*β* ± SE (95% CI)	*p*‐Value
Sex (male)	3.700 ± 1.618 (0.505, 6.895)	.024	4.014 ± 1.725 (0.604, 7.425)	.021
Age (years)	−0.172 ± 0.054 (−0.278, −0.066)	.002	−0.091 ± 0.061 (−0.212, 0.030)	.141
Diabetes	−4.961 ± 1.612 (−8.144, −1.778)	.002	−4.214 ± 1.703 (−7.580, −0.847)	.015
Albumin (g/L)	0.833 ± 0.226 (0.386, 1.28)	<.001	0.514 ± 0.238 (0.043, 0.985)	.033
Creatinine (μmol/L)	0.008 ± 0.003 (0.003, 0.013)	.003	−0.001 ± 0.004 (−0.008, 0.008)	.990
Phosphorus (mmol/L)	3.525 ± 1.705 (0.158, 6.893)	.040	1.132 ± 1.961 (−2.744, 5.009)	.564
HDL‐C (mmol/L)	5.565 ± 2.851 (−0.067, 11.197)	.053	4.792 ± 3.125 (−1.386, 10.970)	.127
LDL‐C (mmol/L)	2.571 ± 1.010 (0.575, 4.567)	.012	0.206 ± 1.027 (−1.824, 2.237)	.841
Hs‐CRP (mg/L)	−0.316 ± 0.162 (−0.637, 0.004)	.053	0.061 ± 0.163 (−0.261, 0.384)	.708
NLR	−1.529 ± 0.411 (−2.34, −0.718)	<.001	−1.208 ± 0.585 (−2.364, −0.052)	.041
PLR	−0.017 ± 0.012 (−0.041, 0.006)	.149	0.021 ± 0.014 (−0.008, 0.049)	.150
MLR	−14.152 ± 3.465 (−20.994, −7.309)	<.001	−8.113 ± 4.895 (−17.792, 1.566)	.100

Abbreviations: 25(OH)D, 25‐hydroxyvitamin D; CI, confidence interval; HDL‐C, high‐density lipoprotein cholesterol; hs‐CRP, high‐sensitivity C‐reactive protein; LDL‐C, low‐density lipoprotein cholesterol; MLR, monocyte to lymphocyte ratio; NLR, neutrophil to lymphocyte ratio; PLR, platelet to lymphocyte ratio.

## 4 DISCUSSION

Vitamin D is a fat‐soluble steroid and plays a central role in bone and mineral metabolism through its hormonal form 1,25‐dihydroxyvitamin D, which is produced by 1‐alfa hydroxylase in the kidney. Recent data revealed a notably wide tissue distribution of vitamin D receptor and 1‐alfa hydroxylase in the same or neighboring cells.^12^ Decreased circulating 1,25‐dihydroxyvitamin D is common in ESRD patients due to the loss of renal 1‐alfa hydroxylase. Thus, the external pathway enabling the transformation of 25(OH)D into 1,25‐dihydroxyvitamin D via 1‐alfa hydroxylase is a contributing factor for many nonclassical actions of vitamin D which takes effect in ESRD patients. It is estimated that over 85% of serum 25(OH)D is utilized by local target tissues for autocrine and paracrine activation of 1,25‐dihydroxyvitamin D,^13^ thus impacting on inflammatory process in different tissues.^14^ Besides, a previous study showed that vitamin D deficiency is a highly prevalent and modifiable risk factor in patients with CKD, contributing to inflammation.^15^ Therefore, vitamin D supplementation is pivotal for patients with ESRD.

A study has confirmed the significant association between serum 25(OH)D level with age, diabetes mellitus, albumin level, and creatinine level. The linear regression analysis results proved that sex, diabetes mellitus, and albumin level are independently related to 25(OH)D level, while age, sex, race, diabetes, current smoking, and BMI are all independently associated with 25(OH)D deficiency in the general population.^16^ Age^17^ and diabetes^18^ are the determinants for predicting 25(OH)D level in CKD patients. In hemodialysis‐treated patients, it was documented that 25(OH)D level is linked to age, sex, and diabetes but not to albumin level.^19^, ^20^ A previous report showed that patients with a decreased 25(OH)D level have a lower albumin level, but the difference was not significant.^21^ Bansal et al.^22^ found a weak correlation between 25(OH)D level with weight, sex, levels of hemoglobin, albumin, and alkaline phosphatase, and diabetes in hemodialysis‐treated patients. All these studies of dialysis‐treated patients did not mention the relationship between vitamin D level and creatinine level. Yildirim et al.^23^ reported that creatinine level is markedly lower in 25(OH)D‐deficient patients without CKD than in patients with normal 25(OH)D level without CKD. In the present study, low creatinine level was observed in 25(OH)D‐deficient patients with statistically significant difference. The associations between vitamin D with levels of albumin and creatinine confirmed poor nutrition in vitamin D‐deficient patients.

CRP is the most common marker to measure inflammation in CKD and ESRD patients. The relationship between vitamin D and CRP is still controversial. Yildirim et al.^23^ studied the associations between 25(OH)D level with CRP, WBC, and erythrocyte sedimentation rate in the general population and nondialysis‐treated CKD patients, and found no relationship between 25(OH)D level and CRP. Several other studies of hemodialysis‐treated patients indicated that 25(OH)D level is inversely correlated with CRP.^19^, ^21^, ^24^ In contrast to these studies, although the median of CRP values was higher in patients with 25(OH)D deficiency in our study, the difference was not statistically significant and there was no prominent correlation between CRP and 25(OH)D level, which may be due to individual characteristics of the studied population. The values of CRP in our cohort were much lower compared to the studies mentioned above. Intriguingly, NLR, PLR, and MLR were all positively correlated with CRP in our study. Furthermore, in terms of inflammation determination, either NLR^25^, ^26^ or PLR^27^, ^28^ has been confirmed to be the superior one by different studies. CRP in combination with NLR can reduce the CRP cutoff point for distinguishing between infectious and noninfectious inflammation in hemodialysis‐treated patients.^29^ Our study unveiled that NLR had a stronger correlation with CRP than PLR and MLR. NLR might be better for identifying inflammation in hemodialysis than PLR and MLR.

NLR, PLR, and MLR are not only used to determine inflammation,^30^, ^31^, ^32^ but also considered to be predictors of all‐cause and cardiovascular mortality^33^, ^34^, ^35^, ^36^, ^37^, ^38^, ^39^ in ESRD patients and dialysis‐treated patients. Numerous studies have compared the roles of NLR, PLR, and MLR in predicting cardiovascular and all‐cause mortality in hemodialysis‐treated patients. Catabay et al.^40^ found that NLR, instead of PLR, is a mortality predictor. Yaprak et al.^28^ reported that both NLR and PLR are associated with all‐cause mortality in hemodialysis‐treated patients but only PLR can independently predict all‐cause mortality. Zhang et al.^41^ identified that the high NLR value is associated with high all‐cause mortality while PLR is a predictor of cardiovascular mortality. Xiang et al.^42^ unraveled that high value of MLR strongly and independently predicts all‐cause and cardiovascular mortality compared to NLR.

The relationship between NLR and PLR with vitamin D in hemodialysis‐treated patients has been less studied. In line with previous data, NLR is obviously higher in the hemodialysis‐treated patients with 25(OH)D < 10 ng/mL and is inversely correlated with 25(OH)D level.^43^ Kara et al.^19^ found that NLR is evidently higher in the 25(OH)D deficiency group in comparison with the normal group. Serum 25(OH)D level is inversely correlated with NLR and PLR, but when determined by linear regression analysis, sex is the only variable significantly associated with 25(OH)D level. In Villafuerte‐Ledesma et al.'s^21^ study, NLR and PLR are both higher in the presence of 25(OH)D deficiency, manifesting the inverse correlation between NLR and PLR with 25(OH)D level. There was no study investigating the interplay between 25(OH)D and MLR in hemodialysis‐treated patients. Our study corroborated that NLR and MLR were both noticeably higher in hemodialysis‐treated patients with 25(OH)D deficiency and had a statistically significant negative correlation with 25(OH)D level. Moreover, multiple linear regression analysis results verified that NLR was independently associated with serum 25(OH)D level after adjustments for other factors. Thus, the function of NLR was superior to those of MLR and PLR in our study. However, the mechanism of decreasing inflammatory when serum 25(OH)D levels still unclear. Previous study showed that 25(OH)D affects proinflammatory markers IL‐1β, IL‐6, IL‐8, TNF‐α via nuclear factor kappa‐B and mitogen‐activated protein kinase signaling pathways.^44^ Moreover, 25(OH)D inhibited B‐cell activation and immunoglobulin synthesis, reduced Th1 and Th17 cells and the production of IFN‐γ, IL‐17, IL‐6, IL‐23, and IL‐2, and increases Th2 cells, thereby mediating cytokines and TNF‐α to limit the inflammatory process.^45^ Base on this, it is reasonable to suspect that the possible role of 25(OH)D in ESRD can be explained by its role in the regulation of immune cells and inflammatory pathways, which need more experiments to verify.

To the best of our knowledge, this is the first study to determine the relationship between 25(OH)D with MLR, NLR, and PLR in hemodialysis‐treated patients. Nonetheless, our study has several limitations. First, this was a cross‐sectional study which cannot establish a causal relationship between serum 25(OH)D level and inflammatory markers. Second, biochemical measurements were only performed once. Time‐ and season‐dependent changes of 25(OH)D level were not investigated. Thirdly, the impact of therapeutic interventions was not considered. Lastly, the sample size was relatively small, although the number of patients was adequate to show the statistical significance.

## 5 CONCLUSIONS

Our study demonstrates an inverse association of serum 25(OH) D level and inflammatory markers. Further, large‐scale, prospective, and interventional studies are needed to confirm our results and to deeply investigate the associations between serum 25(OH) D level with inflammatory markers in hemodialysis‐treated patients.

## AUTHOR CONTRIBUTIONS

Chunlei Luo and Xueyan Bian performed the research. Chunlei Luo designed the research study. Lingling Bao, Qingqing Xu, and Chunyang Ji contributed essential reagents or tools and analyzed the data. Chunlei Luo, Xueyan Bian, Lingling Bao, Qingqing Xu, and Chunyang Ji wrote the paper, read and approved the final manuscript.

## CONFLICT OF INTEREST STATEMENT

The authors declare no conflicts interest.

## ETHICS STATEMENT

The data of 167 patients who met the criteria were included in the study, which was approved by the ethics committee of our hospital. Written informed consent was obtained from the patient for publication.

## Data Availability

The analyzed data sets generated during the study are available from the corresponding author on reasonable request.
